# A Silent Threat: Internal Carotid Artery Hypoplasia and Its Role in Basilar Artery Aneurysm Formation—A Case Study

**DOI:** 10.3390/diagnostics15060774

**Published:** 2025-03-19

**Authors:** Paula Mežvinska, Artis Brokāns, Sergejs Pavlovičs, Matīss Dravnieks, Ardis Platkājis, Arturs Balodis

**Affiliations:** 1 Faculty of Medicine, Riga Stradins University, 16 Dzirciema Street, LV-1007 Riga, Latvia; 2 Department of Radiology, Riga Stradins University, 16 Dzirciema Street, LV-1007 Riga, Latviaardis.platkajis@rsu.lv (A.P.); 3 Institute of Diagnostic Radiology, Pauls Stradins Clinical University Hospital, 13 Pilsonu Street, LV-1002 Riga, Latvia; 4 Department of Neurosurgery, Pauls Stradins Clinical University Hospital, 13 Pilsonu Street, LV-1002 Riga, Latvia; 5 Department of Radiology, Riga First Hospital, 5 Bruņinieku Street, LV-1001 Rīga, Latvia

**Keywords:** internal carotid artery hypoplasia, ophthalmic artery, basilar artery aneurysm, subarachnoid hemorrhage

## Abstract

**Background and Clinical Significance:** Hypoplasia of the internal carotid artery (ICA) is a rare vascular anomaly, with an estimated incidence of less than 0.01%. This condition can result in reduced blood flow to the anterior circulation, often compensated by collateral circulation. Radiological imaging, particularly computed tomography angiography (CTA), digital subtraction angiography (DSA), magnetic resonance angiography (MRA), and ultrasound, plays a crucial role in diagnosing this condition, revealing structural abnormalities in the arterial system. **Case Presentation:** This case is about a 75-year-old woman who lived her entire life unaware of ICAH until a basilar artery aneurysm ruptured, leading to a large, centrally localized SAH. Further diagnostic workup, including CTA and DSA, confirmed left ICA hypoplasia, with the artery terminating as the ophthalmic artery, along with a developmental anomaly of the left middle cerebral artery from posterior circulation territory. Due to the high risk associated with surgical and endovascular intervention, conservative management was pursued, and the patient’s condition stabilized, though she continued to have significant neurological deficits. **Conclusions:** This case report supports the hypothesis that ICAH may be associated with aneurysm formation. This case demonstrates that if ICAH is not diagnosed early, it can lead to severe complications with permanent neurological deficits. Additionally, it highlights the critical importance of advanced imaging techniques, such as CTA and DSA, in diagnosing complex vascular conditions.

## 1. Introduction

Internal carotid artery hypoplasia (ICAH) is a rare congenital vascular anomaly where the development of the internal carotid artery (ICA) is incomplete or arrested. This condition is defined by a notable narrowing of the ICA lumen, typically occurring 1–2 cm above the carotid bifurcation and continuing along the artery’s length [1]. ICAH has an estimated prevalence of less than 0.01%, although incidental findings suggest that the actual rate may be higher [2,3,4,5]. ICAH is one of three main types of ICA dysplasia, with the others being agenesis (complete absence of the ICA) and aplasia (underdevelopment or partial absence of the ICA) [6].

In this paper, we present the case of a patient diagnosed with a ruptured fusiform basilar artery aneurysm, accompanied by uncommon vascular anomalies, such as hypoplasia of the left internal carotid artery and an aberrant middle cerebral artery arising from the posterior circulation. Additionally, this paper includes a brief review of the literature on internal carotid artery hypoplasia to contextualize and better understand this rare but potentially life-threatening condition.

## 2. Case Report

**Figure 1:**
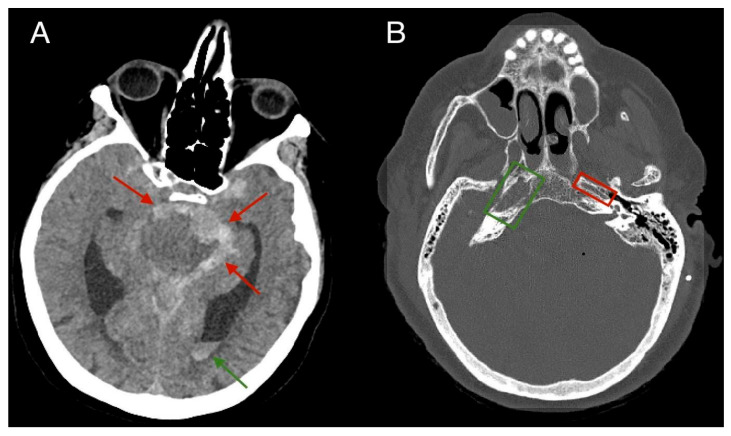
(**A**) Non-contrast CT scan of the brain, axial plane. The scan shows a large subarachnoid hemorrhage (SAH) around the basal cisterns and the circle of Willis (red arrows). An intraventricular hemorrhage (green arrow) and lateral ventricular enlargement—a sign of obstructive hydrocephalus—were also noted. (**B**) CT scan of the brain, axial plane, bone window. The patient’s left carotid canal (red rectangle) is narrowed, suggestive of stenosis, measuring approximately 2.7 mm in D, while the right carotid canal (green rectangle) measures within the average range, around 7.5 mm.

**Figure 2:**
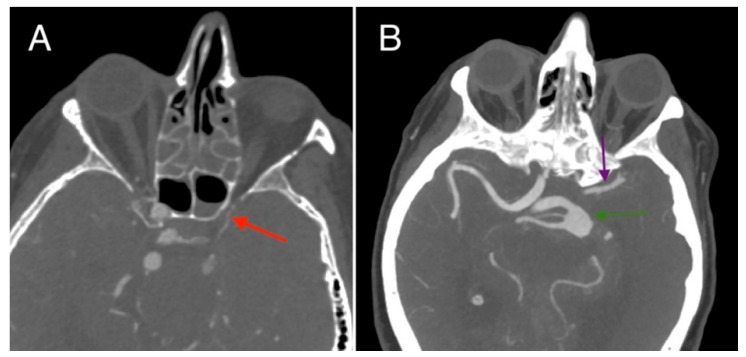
(**A**) Computed tomography angiography (CTA), axial plane. An image demonstrating a hypoplastic left internal carotid artery terminating in the ophthalmic artery (red arrow). (**B**) CTA, axial plane. An image showing a fusiform aneurysm at the apex of the basilar artery measuring approximately 12 × 16 mm (green arrow). The M1 segment of the middle cerebral artery (purple arrow).

**Figure 3:**
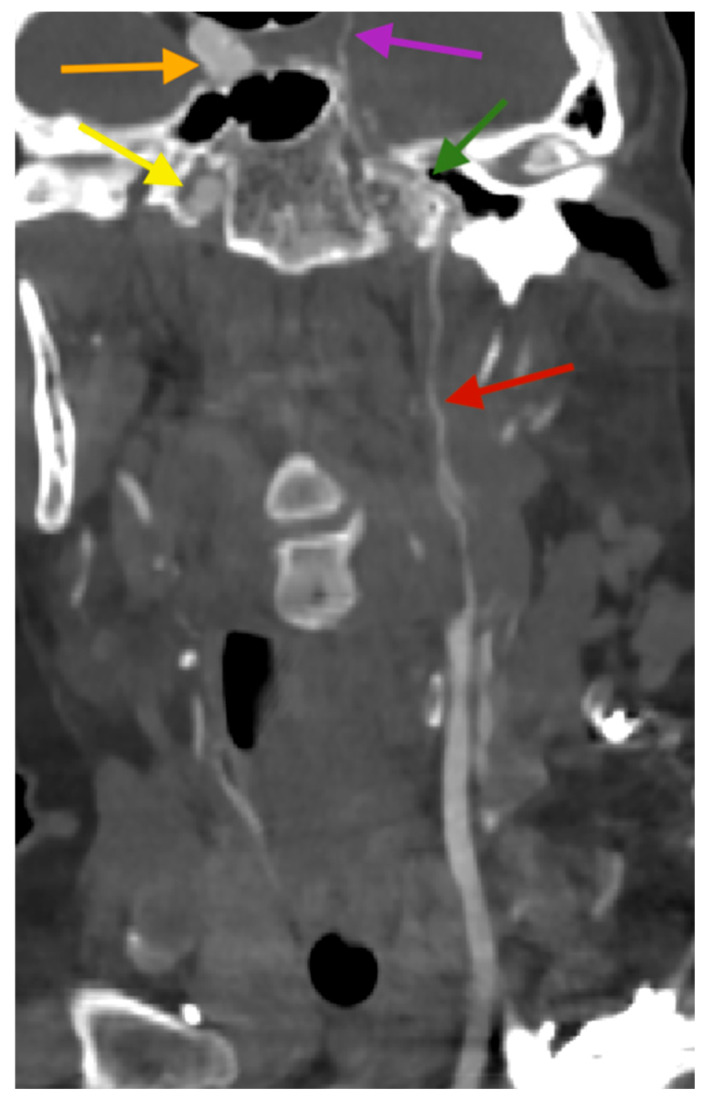
Computed tomography angiography reconstruction, coronal plane. The reconstruction displays a hypoplastic left internal carotid artery along its entire length, including the C1 (cervical part—red arrow), C2 (petrous part—green arrow), and C4 (cavernous part—purple arrow). The normal right internal carotid artery is visible, showing the C2 (petrous part—yellow arrow) and C4 (cavernous part—orange arrow).

**Figure 4:**
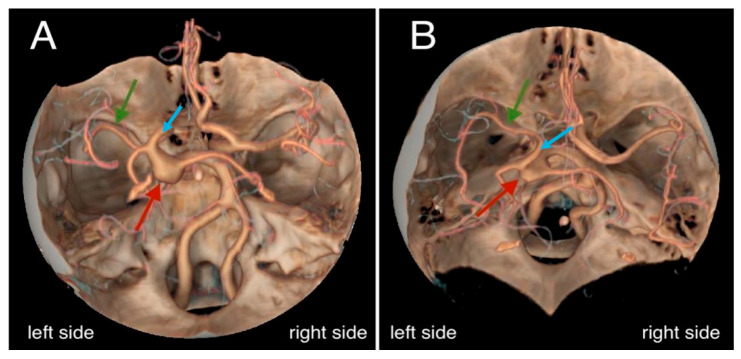
Computed tomography angiography volume rendering (VR) reconstructions. (**A**,**B**) The reconstruction was performed to assess vascular structures, revealing a fusiform aneurysm at the distal end of the basilar artery (red arrows), measuring approximately 12 mm in diameter (⌀). The middle cerebral artery (MCA), identified by green arrows, appears to arise from a bifurcation through a fetal-type posterior communicating artery (Pcom) (blue arrows), rather than directly from the basilar artery. This configuration is rare, with the MCA divided into its typical anatomical segments (M1 through M4), supplying the expected MCA territory.

A 75-year-old female patient, with a history of hypertension, communicated with her son via phone at approximately 3:00 a.m., during which she had no complaints. Later that morning, she developed impaired consciousness and was hospitalized. Upon admission, a hypertensive crisis was identified. The patient had a Glasgow Coma Scale (GCS) score of 10, with anisocoria noted (right pupil smaller than the left). A non-contrast CT scan of the head revealed a large subarachnoid hemorrhage (SAH) in the region of the basal cisterns and around the circle of Willis. The bone window of the CT scan demonstrated narrowing of the left carotid canal, measuring approximately 2.7 mm in diameter, compared to the right carotid canal, which measured around 7.5 mm in diameter. Signs of obstructive hydrocephalus were also noted (Figure 1). Subsequent CT angiography (CTA) demonstrated a fusiform aneurysm of the basilar artery with evidence of rupture, a hypoplastic left internal carotid artery terminating in the ophthalmic artery, and an abnormal origin of the middle cerebral artery (MCA), which appears to arise through a fetal-type posterior communicating artery (Pcom) rather than directly from the basilar artery, resulting in an aberrant MCA (Figure 2). Further CTA reconstructions displayed a hypoplastic left internal carotid artery throughout its entire length, including all segments (Figure 3). Further volume reconstruction (VR) displayed that the aberrant MCA, originating from the posterior circulation, is further divided into its anatomical segments (M1 through M4) and supplies the typical MCA territory (Figure 4).

**Figure 5:**
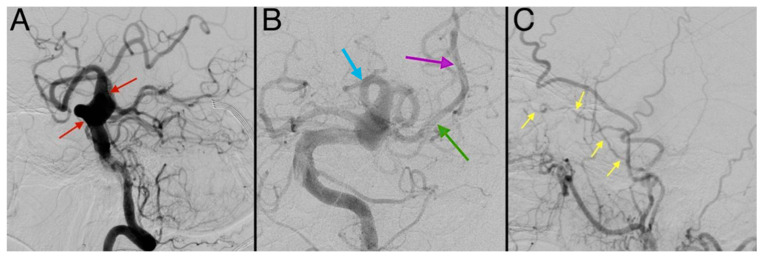
Digital subtraction angiography (DSA) images. (**A**) An image showing an elongated and ectatic basilar artery with a fusiform aneurysm (red arrows) at its distal end, measuring approximately 10 mm in diameter (⌀). (**B**) An image demonstrating the middle cerebral artery (M1 sphenoidal segment—green arrow; M2 insular segment—purple arrow) arising through a fetal-type posterior communicating artery (Pcom) (blue arrow). (**C**) An image displaying a hypoplastic left internal carotid artery terminating in the ophthalmic artery (yellow arrows).

The following day, digital subtraction angiography (DSA) confirmed findings observed on CTA: an anomaly in the development of the left middle cerebral artery, hypoplasia of the left internal carotid artery with termination in the ophthalmic artery, and a ruptured fusiform basilar artery aneurysm (Figure 5). Due to obstructive hydrocephalus, an external ventricular drain (ventriculostomy) was placed. Post-procedure, the patient was transferred to the intensive care unit (ICU) for mechanical ventilation, sedoanalgesia, and hemodynamic stabilization.

She remained in a post-anesthetic state with anisocoria (right pupil smaller than the left). Sedoanalgesia was maintained with intravenous propofol and fentanyl. For vasospasm prophylaxis, a continuous nimodipine infusion was set at 1–2 mg/h. Blood pressure was managed with low-dose norepinephrine to maintain a systolic blood pressure above 120 mmHg and a mean arterial pressure (MAP) of 85 mmHg. Cerebrospinal fluid drainage from the ventriculostomy averaged approximately 280 mL per day. Mechanical ventilation was continued in pressure-controlled modes with FiO_2_ of 0.4 and PEEP of 7 cmH_2_O. Hemodynamic parameters were stable, with a blood pressure of 145/57 mmHg, heart rate of 68 bpm, and intracranial pressure (ICP) of 9 mmHg. Enteral feeding was initiated in minimal amounts via a nasogastric tube. 

One week later, a trial of external ventricular drain (EVD) weaning was carried out by raising the EVD threshold to 20 mmH_2_O for 8 h and later clamping the drain for 4 h. On a CT scan, hydrocephalus was visible, and the patient’s GCS score declined, necessitating ventriculoperitoneal shunting. A tracheostomy was also performed two days later. A multidisciplinary case review, involving neurosurgeons and interventional radiologists, concluded that invasive treatment of the aneurysm posed a prohibitively high risk of clinical deterioration and mortality, given the patient’s overall condition, her neurological status, and the aneurysm’s location, morphology, and size. The patient’s NIHSS (National Institutes of Health Stroke Scale) score was 20, indicating a severe neurological deficit, and the mRS (modified Rankin scale) was 5, reflecting significant disability with severe functional dependence. Given the patient’s neurological condition, with a GCS score of 8, endovascular treatment, including the insertion of a flow diverter, was deemed unsuitable and not performed. Conservative management was, therefore, recommended.

**Figure 6:**
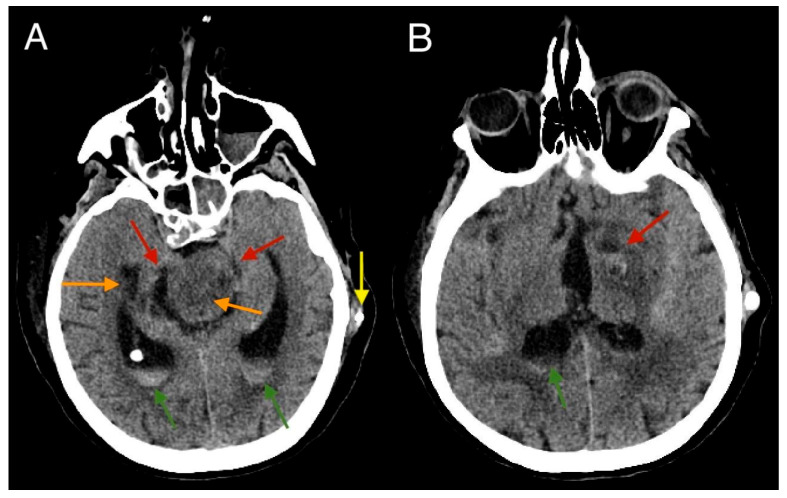
A follow-up CT scan performed 15 days after the initial scan (**A**,**B**) indicating a slight resolution of the subarachnoid hemorrhage (red arrows). Partially residual hemorrhagic content is visible in both lateral ventricles (green arrow). A shunt from the external ventricular drainage can also be noted (yellow arrow). Post-ischemic changes at the level of the pons and left basal ganglia can also be observed (orange arrows).

A follow-up CT scan performed 15 days after the initial scan showed a slight resolution of the subarachnoid hemorrhage. Partially degraded hemorrhagic content could be observed in both lateral ventricles (Figure 6).

Over time, the patient’s condition gradually stabilized, and she was transferred to a lower-level care facility. Upon discharge, her neurological status was as follows: GCS score of E3, Vt (tracheostomy), M6, with persistent anisocoria (right pupil smaller than the left), right-sided plegia, and severe left-sided paresis (muscle strength 4/5, arm more affected than leg).

## 3. Discussion

ICAH is known to predominantly present unilaterally, with the left side more frequently affected [7,8]. Similarly, in our case, the patient exhibited ICAH on the left side, aligning with this observed trend.

The exact mechanisms behind this vascular anomaly are not fully understood. According to one prominent theory, ICAH results from incomplete formation of the fetal dorsal aorta during early embryonic development. Developmental abnormalities in vascular morphogenesis, particularly during the fourth to sixth weeks of embryonic development, are the root cause of ICAH. During this period, the dorsal aorta and third aortic arch play a crucial role in ICA development. The left ICA appears more commonly affected due to the increased complexity of vascular development on this side, which includes the formation of the aortic arch [7]. Hemodynamic factors, such as variations in blood flow patterns during fetal development, may further contribute to asymmetry, making the left ICA more susceptible to hypoplasia [7]. Disturbances in the formation of these structures can result in underdeveloped ICAs, reducing their ability to supply the anterior brain circulation. To compensate, collateral circulation is established, mainly involving the posterior communicating artery (PComA) and the basilar artery. These compensatory mechanisms alter cerebral blood flow, creating increased stress on the collateral vessels. Over time, this can lead to aneurysm formation, particularly in the basilar artery, and may result in conditions like subarachnoid hemorrhage (SAH), as seen in our case [9,10]. While we hypothesize that hemodynamic changes related to these alterations in circulation could contribute to aneurysm formation, we do not have direct evidence to confirm that hemodynamics were the definitive cause in this instance. Individuals with ICA hypoplasia are at a significantly higher risk for developing intracranial aneurysms, with an incidence rate of 27.8%, which is far higher than the 2–4% seen in the general population [11]. However, other factors can also contribute to aneurysm formation, including hypertension, smoking, insomnia, and genetic predisposition [12,13]. Additionally, some genetic syndromes, including DiGeorge syndrome, neurofibromatosis 1 and 3, and pseudoxanthoma elasticum, can be associated with ICAH [14,15,16]. 

A particularly rare variant of ICAH involves the artery terminating prematurely as the ophthalmic artery. This anomaly disrupts the anterior circulation, forcing posterior circulation vessels to adapt. These hemodynamic adaptations, while compensatory, place undue stress on vessels such as the basilar artery, heightening the risk of aneurysm formation. Cases like these underscore the intricate interplay between congenital anomalies and compensatory vascular mechanisms [11,17]. For instance, Türk et al. reported a patient whose hypoplastic ICA terminated as the ophthalmic artery, accompanied by saccular aneurysms in the contralateral ICA. The authors attributed these aneurysms to hemodynamic stresses arising from the hypoplastic artery’s inability to contribute to normal cerebral blood flow [18].

In 1968, Lie et al. developed a system to classify ICA variants based on patterns of collateral circulation, identifying six types (A–F). Although certain cases corresponded to type E, many others could not be classified within this system, leading some researchers to propose new categories or highlight overlapping characteristics [19]. Later, Zhang and colleagues introduced a classification centered on the ophthalmic segment, examining 20 cases of ICAH. They divided the anomalies into two groups: 60% involving occlusion of the ophthalmic segment and 40% involving non-ophthalmic segment occlusion. The former had fewer ischemic events, likely due to adequate PComA circulation, whereas the latter experienced more ischemic events with varied collateral patterns. Further studies are needed to validate this classification system [20].

In the case we report, the patient remained asymptomatic regarding her ICAH throughout her life. It was only at age 75, following the rupture of a basilar artery aneurysm, that the condition was discovered. The patient found out about her hypoplastic ICA only during her hospitalization following the rupture, when diagnostic imaging studies, including computed tomography angiography (CTA) and digital subtraction angiography (DSA), were performed. This case highlights the silent and often asymptomatic nature of ICAH, as the condition frequently remains undiagnosed until secondary complications, such as aneurysm formation or rupture, arise. Most patients with ICAH are diagnosed incidentally when imaging is performed for unrelated reasons. For example, Zink et al. reported that, in their study, 39 out of 165 cases of ICA aplasia or hypoplasia were identified incidentally [8].

The lack of symptoms is typically due to the development of sufficient collateral circulation, which compensates for the reduced blood flow through the hypoplastic ICA. However, when symptoms do occur, they can vary widely, ranging from headaches [21] and transient ischemic attacks (TIAs) [22] to hemiplegia [23], dysarthria [24], and vision loss [25]. The severity of symptoms is closely linked to the adequacy of collateral circulation. A ruptured aneurysm causing subarachnoid hemorrhage is one of the most serious complications of ICAH, emphasizing the importance of early recognition and management of this condition [26].

Advanced imaging modalities are essential in diagnosing ICAH. Computed tomography angiography (CTA) and magnetic resonance angiography (MRA) are effective for visualizing ICA anomalies, while digital subtraction angiography (DSA) remains the gold standard for assessing vascular morphology and flow dynamics [27]. A key diagnostic feature is the diameter of the carotid canal, which reflects ICA development during embryogenesis. Hypoplastic ICAs typically correspond to carotid canals measuring less than 3 mm in diameter, distinguishing them from stenotic ICAs, which have normal canal sizes [28]. The mean diameters of hypoplastic carotid canals in the axial (transverse diameter) and sagittal (height) views were 3.1 and 3.3 mm, respectively, whereas on the normal side, the mean diameters were 5.4 and 5.6 mm [29]. In our reported case, the diameter of the carotid canal on the left side was approximately 2.7 mm, compared to 7.5 mm on the right side. Agenesis, in contrast, is marked by the absence of a carotid canal altogether. The development of the internal carotid artery during embryogenesis is closely associated with the formation of the carotid canal, and a reduced canal size suggests a developmental anomaly [2]. A bone-targeted CT of the skull base is considered a reliable method for evaluating the carotid canal and plays a vital role in diagnosing ICAH [1].

In addition to carotid canal size, the internal carotid artery lumen diameter also provides valuable diagnostic information. The typical diameter of the internal carotid artery lumen is around 5 mm [30], although this measurement is not routinely performed in clinical practice. Zhang et al., in a retrospective analysis of 20 patients with congenital ICAH, found the mean lumen diameter of hypoplastic ICAs to be 1.6 mm, with a range of 1.4 to 1.9 mm [20]. Chen et al. highlighted the role of color-coded carotid duplex sonography in identifying hypoplastic ICA features, such as a reduced lumen diameter and decreased flow volume, while excluding intraluminal atherosclerosis or other structural abnormalities [29]. Combining these sonographic findings with advanced imaging techniques such as CTA, MRA, and skull base CT ensures a comprehensive evaluation of ICAH and associated vascular anomalies. 

Acquired stenosis, dissection, fibromuscular dysplasia, Moyamoya disease, and occlusion secondary to atherosclerosis are among the diseases that are included in the differential diagnosis for ICAH [31]. Imaging is essential for differentiating between these conditions. Fibromuscular dysplasia, for instance, typically shows a “string of beads” pattern, while stenosis and atherosclerotic changes show wall irregularities or calcifications with normal carotid canal diameters [32]. ICA dissection is identified by imaging features such as an intimal flap or double-lumen appearance [33]. Differentiating ICAH from Moyamoya disease can be challenging, as both conditions involve narrowed carotid canals. However, angiography reveals distinct characteristics: in ICAH, a narrowing of the ICA occurs 1–2 cm above the bifurcation, whereas Moyamoya disease involves progressive narrowing or occlusion of the distal ICA and the proximal portions of the middle and anterior cerebral arteries [34]. 

Currently, there are no specific guidelines for managing ICAH. Asymptomatic patients may only require conservative management with periodic imaging. However, due to increased risk of aneurysms and cerebrovascular insufficiency, it is important to control blood pressure, cholesterol, and smoking. Prompt imaging is advised for patients with neurological symptoms. Unruptured aneurysms are often treated with endovascular techniques like coiling or stent-assisted procedures. In the event of rupture, as seen with SAH, emergent interventions, including aneurysm occlusion and cerebrospinal fluid diversion to manage hydrocephalus, are necessary [21,31,35].

The limitations of this case report include the lack of long-term follow-up data, which restricts the ability to assess the patient’s recovery and prognosis. Additionally, the literature review included in this report is based on a relatively small number of cases, limiting the generalizability of the findings. The inadequate sample size in the literature makes it challenging to establish firm conclusions regarding the clinical course, management strategies, and outcomes for patients with ICAH.

## 4. Conclusions

In this instance, ICAH involved the artery terminating prematurely as the ophthalmic artery, further complicating cerebral circulation and heightening the risk of aneurysm formation. The diagnosis was made when the patient was 75 years old, illustrating how ICAH can remain undetected for decades due to its gradually appearing complications. Often, the condition only becomes evident after a critical event, such as an aneurysm rupture. This case contributes to the hypothesis that ICAH may be associated with aneurysm formation; however, further studies are needed to confirm this association. Further research with larger case studies is needed to better understand the long-term outcomes of ICAH and its association with cerebrovascular events, as well as to refine diagnostic and management strategies for these challenging cases.

## Data Availability

The data presented in this study are available upon request from the corresponding author. The data are not publicly available due to privacy restrictions.
